# Supplementary material for: Content-rich biological network constructed by mining PubMed abstracts
Source: BMC Bioinformatics. 2004 Oct 8;5:147. doi: 10.1186/1471-2105-5-147 (PMC528731; doi:10.1186/1471-2105-5-147)
Supplement: Additional File 2 — The original results of the above study (non-essential files are deleted to keep the file size under the limit set by BMC bioinformatics). [file 1471-2105-5-147-S2.bz2 › chilibotAdditionalFile2/dip10/213ID7790364E719/html/ITGA3_CD9.html]

 


 **ITGA3** and **CD9** 
  
Found 15 abstracts in PubMed, retrieved 10.  
 

 What does Google say? 
 PDF only 
| .edu only 

---

**Interactive relationship** (e.g. stimulation, inhibition, etc)

**Non-interactive relationship** (e.g. studied together, co-existance, homology, etc.)

- The loss of MRP1  [ **CD9** ]   **CD9**  and integrin alpha3  [ **ITGA3** ]  has been involved in tumor growth and metastasis of cancer cells.  Ref: 11505398 Cancer, 2001
- The aim of the current study was to clarify the clinical significance of MRP1  [ **CD9** ]   **CD9**  and integrin alpha3  [ **ITGA3** ]  in endometrial cancer.  Ref: 11505398 Cancer, 2001
- Data obtained are presented in concurrence with Clusters of Thymic Epithelial Staining CTES classification for various monoclonal antibodies recognizing CD antigens CD1, CD1a, CD6,  **CD9** , CD14, CD16, CD29, CD30, CD32, CD44, CD45RB, CD47, CD48, CD49a, CD49b, CD49c  [ **ITGA3** ] , CD49d, CD49e, CD49f, CD51, CD53, CD54, CD56, CD57, CD63, CD85, CD95, CD98, CD102, CD103, CD106, CD109, CD146, CD147, CD148, CD151, CD152, CD158a, CD158b, CD164, CD165, CD166 and for monoclonal antibodies 1B1 G7, A4, BD46, BLTZ, HP1C5, IND.64, M72, WU947 whose specifities are not yet defined.  Ref: 12725440 Ann Anat, 2003
- Integrin alpha3  [ **ITGA3** ]  expression as a prognostic factor in colon cancer association with MRP 1  [ **CD9** ]   **CD9**  and KAI1 CD82.  Ref: 11802216 Int J Cancer, 2002
- RESULTS HB EGF, its receptors HER 1 and HER 4, and the associated proteins  **CD9** , CD44, and integrin alpha 3  [ **ITGA3** ]  beta 1 were expressed by HPMCs and peritoneal macrophages.  Ref: 11168942 Kidney Int, 2001
- Loss of motility related protein  [ **CD9** ]  1 MRP1  [ **CD9** ]   **CD9**  and integrin alpha3  [ **ITGA3** ]  expression in endometrial cancers.  Ref: 11505398 Cancer, 2001
- RESULTS In normal endometrium, MRP  **CD9**  was expressed at the cell membrane of cell contact sites, and the expression of integrin alpha3  [ **ITGA3** ]  was detected also at the cell membrane of cell contact sites and at borders of stromal tissues.  Ref: 11505398 Cancer, 2001
- Disease free survival curves were estimated using the Kaplan Meier method and analyzed by the log rank test between the positive and reduced expression statuses of both MRP1  [ **CD9** ]   **CD9**  and integrin alpha3  [ **ITGA3** ] .  Ref: 11505398 Cancer, 2001
- Immunoprecipitation followed by Western blot analysis showed association between  **CD9**  and integrin alpha3  [ **ITGA3** ]  in endometrium.  Ref: 12021064 Biol Reprod, 2002
- CONCLUSIONS These findings suggested that the analysis for the expression statuses of MRP1  [ **CD9** ]   **CD9**  and integrin alpha3  [ **ITGA3** ]  may provide important information on the clinical behavior of endometrial cancer.  Ref: 11505398 Cancer, 2001
- Anti  **CD9** , CD26, CD29, CD31, CD36, CD44, CD49a, CD49b, CD49c  [ **ITGA3** ] , CD49d, CD49e, CD49f, CD54, CD55, CD56, CD62E &.  Ref: 10235320 J Pediatr Surg, 1999
- METHODS The expression of MRP1  [ **CD9** ]   **CD9**  and integrin alpha3  [ **ITGA3** ]  from the same tissue sample were examined immunohistochemically in 15 patients with normal endometrium and in 56 patients with uterine endometrioid adenocarcinoma.  Ref: 11505398 Cancer, 2001
- Fourteen cases indicated a reduced expression of both MRP1  [ **CD9** ]   **CD9**  and integrin alpha3  [ **ITGA3** ] .  Ref: 11505398 Cancer, 2001
- A correlation was found between integrin alpha3  [ **ITGA3** ]  with MRP 1  [ **CD9** ]   **CD9**  and KAI1 CD82 for stage I tumors.  Ref: 11802216 Int J Cancer, 2002
- colon cancer progresses following collapse of the complex formed by integrin alpha3  [ **ITGA3** ]  with MRP 1  [ **CD9** ]   **CD9**  and KAI1 CD82.  Ref: 11802216 Int J Cancer, 2002
- In patients with endometrioid adenocarcinoma, 17 cases showed reduced expression of MRP1  [ **CD9** ]   **CD9** , and 20 cases had reduced expression of integrin alpha3  [ **ITGA3** ] .  Ref: 11505398 Cancer, 2001
- Each reduced expression of MRP1  [ **CD9** ]   **CD9**  or integrin alpha3  [ **ITGA3** ]  was significantly correlated with histologic grade and metastasis.  Ref: 11505398 Cancer, 2001
- BACKGROUND MRP1  [ **CD9** ]   **CD9**  and integrin alpha3  [ **ITGA3** ]  have played crucial roles in cell adhesion, motility, and signaling events.  Ref: 11505398 Cancer, 2001
- Furthermore, we investigated the correlation of integrin alpha3  [ **ITGA3** ]  with MRP 1  [ **CD9** ]   **CD9**  and KAI1 CD82.  Ref: 11802216 Int J Cancer, 2002
